# Macrophage Autophagy and Bacterial Infections

**DOI:** 10.3389/fimmu.2017.01483

**Published:** 2017-11-06

**Authors:** Aïcha Bah, Isabelle Vergne

**Affiliations:** ^1^Institut de Pharmacologie et de Biologie Structurale, UMR 5089 CNRS—Université de Toulouse, Toulouse, France

**Keywords:** autophagy, macrophage, bacteria, pathogen, phagocytosis, xenophagy, inflammation

## Abstract

Autophagy is a well-conserved lysosomal degradation pathway that plays key roles in bacterial infections. One of the most studied is probably xenophagy, the selective capture and degradation of intracellular bacteria by lysosomes. However, the impact of autophagy goes beyond xenophagy and involves intensive cross-talks with other host defense mechanisms. In addition, autophagy machinery can have non-canonical functions such as LC3-associated phagocytosis. In this review, we intend to summarize the current knowledge on the many functions of autophagy proteins in cell defenses with a focus on bacteria–macrophage interaction. We also present the strategies developed by pathogens to evade or to exploit this machinery in order to establish a successful infection. Finally, we discuss the opportunities and challenges of autophagy manipulation in improving therapeutics and vaccines against bacterial pathogens.

## Introduction

Macroautophagy, hereafter referred to as autophagy, is a lysosomal degradative process that participates in cellular homeostasis by enabling the removal of defective organelles, protein aggregates, or intracellular microorganisms (1). The process is highly regulated by multiple signaling pathways and orchestrated by more than 30 autophagy-related (Atgs) proteins organized in several functional units (2). Upon autophagy activation, Atgs, serine/threonine kinase ULK1, and Beclin-1, in association with Atg14 and type III phosphatidylinositol 3-kinase Vps34, promote the formation of a cup-shaped isolation membrane to engulf the cargo (1). Through concomitant activity of two ubiquitin-like conjugation systems, the covalent linkage of Atg12 with Atg5/Atg16L1 and LC3 lipidation with phosphatidylethanolamime, the isolation membrane elongates into a double-membrane vesicle, called autophagosome. The autophagosome then fuses with lysosomes to form an autolysosome in which the engulfed cargo is degraded. This latter step is mediated by a second Beclin-1 complex, lysosomal-associated membrane protein 1 (LAMP1), and a fusion machinery including SNARE syntaxin-17.

In addition to its role in cellular homeostasis, autophagy is essential to immunity. The autophagy machinery targets intracellular pathogens for degradation, modulates inflammation, and participates in adaptive immune responses (3–5). Here, we review the many functions of autophagy in bacterial infections with a focus on macrophages, the first line of host defenses, and the replicative niche of numerous pathogens.

## Autophagy Machinery in Macrophage Antibacterial Defenses

**Figure 1:**
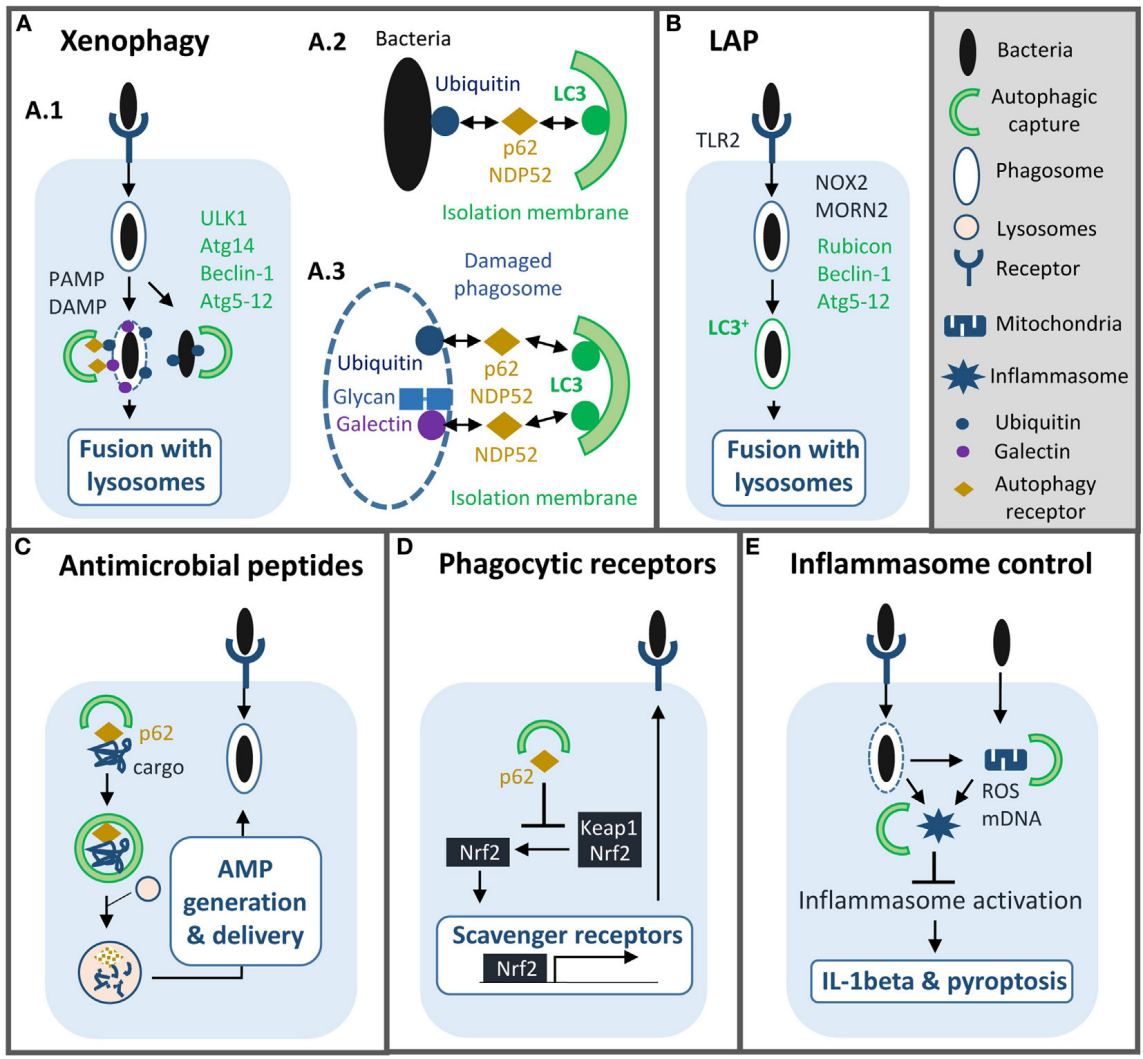
Roles of autophagy machinery in macrophage antibacterial defenses. Autophagy machinery plays several functions in innate immunity to bacterial infection such as: **(A)** xenophagy: selective capture and lysosomal degradation of cytosolic and vacuolar pathogens. Xenophagy requires formation of an autophagosome and depends on ULK1, autophagy-related (Atg)14, Beclin-1, Atg5-12, and autophagy receptor proteins such as p62 (A.1). Bacterial compartment is captured by autophagosome either *via* ubiquitination (A.2, 3) or host glycan recognition by galectins (A.3). **(B)** LC3-associated phagocytosis (LAP): LC3 is conjugated onto the membrane of phagosome containing bacteria to promote fusion with lysosome. LAP depends on Rubicon, Beclin-1, and Atg5-12. **(C)** Induction of autophagy enables production and delivery of antimicrobial peptides to bacterial compartments. **(D)** Autophagy machinery reduces the expression of scavenger receptors to limit phagocytosis of some intracellular pathogens. **(E)** Autophagy controls inflammation by limiting inflammasome activation. PAMP, pathogen-associated molecular pattern; DAMP, damage-associated molecular pattern; AMP, antimicrobial peptide; ROS, reactive oxygen species; mDNA, mitochondrial DNA.

Bacteria induce autophagy mainly *via* their pathogen-associated molecular patterns (PAMPs) and pathogen-induced damage-associated molecular patterns (DAMPs) (4, 5). Cell surface recognition and cytosolic sensing of these molecules result in signaling cascades that promote rapid and localized autophagy machinery recruitment. Autophagy can further be regulated by several transcriptional factors such as NFkappaB and TFEB to promote expression of different autophagy genes and thus prolong autophagy activation (6, 7). Depending on PAMP/DAMP nature and localization, autophagy can selectively capture bacteria, such event is called xenophagy, damaged organelles, and other signaling platforms activated during the infection (4, 5). Furthermore, Atgs proteins have non-autophagic functions essential for innate immunity against bacteria (Figure 1).

### Xenophagy

In macrophages, xenophagy has mainly been characterized during *Mycobacterium tuberculosis* infection but the mechanism is quite similar in epithelial cells infected with *Salmonella typhimurium* (8)*. M. tuberculosis* through its ESX-1 secretion system damages the phagosomal membrane and access the cytosol (9). Cytosolic sensor c-GAS recognizes bacterial DNA, which results in ubiquitination of the bacterium or its phagosome by ubiquitin ligases Parkin and Smurf1 (10–12). Subsequently, ubiquitin chains bind to autophagy adaptors, such as p62 and NDP52, which recruit LC3 to deliver *M. tuberculosis* into an autophagosome. Damaged phagosome can also be targeted by autophagy *via* the recognition of host glycan present on the phagosomal lumen by cytosolic lectins of the galectin family (8, 13). Additionally, in human macrophages, immunity-related GTPase family M protein participates in xenophagy by promoting mitochondrial reactive oxygen species (ROS) production and recruiting autophagy machinery after PAMP exposure (14, 15).

Ultimately, autophagosome sends bacteria to lysosome for degradation (5, 8). In parallel, autophagy can also generate and deliver antimicrobial peptides to bacterial compartment to enhance killing (16, 17). Several, *in vitro*, studies have shown that xenophagy reduces intracellular survival of *M. tuberculosis*, however, its role, *in vivo*, is unclear (9, 12). Mice with monocyte-derived cells and neutrophils lacking Atg5 are more susceptible to *M. tuberculosis*, but not those lacking other Atgs such as Beclin-1 or Atg14, suggesting that autophagy is not involved in controlling the infection (9, 18, 19). Nonetheless, separate studies suggest that autophagy may play a role in a latter chronic phase of infection and/or that *M. tuberculosis* may inhibit the process *in vivo* (12, 20). As we will discuss below, *M. tuberculosis* and other pathogens have developed multiple strategies to block autophagy and some of them have been relevant *in vivo*.

### LC3-Associated Phagocytosis (LAP)

Aside from xenophagy, a non-canonical autophagy process named LAP is known to play an important role in antibacterial defenses (21, 22). Upon phagocytosis, particles or pathogens that engage surface receptors, such as toll-like receptors, Fcgamma receptors or Dectin-1, trigger LC3 conjugation directly onto the phagosomal membrane and independently of autophagosome formation (23–25). In contrast to canonical autophagy, this process does not require ULK1 or Beclin-1/Atg14 complexes but instead relies on Beclin-1/Rubicon complex and NADPH oxidase-2 (NOX2) activation (25, 26). Rubicon activates phosphatidylinositol 3-phosphate (PI3P) synthesis, which in turn, stimulates ROS production. PI3P and ROS then promote recruitment of the two ubiquitin-like conjugation systems to trigger LC3 conjugation. Another protein MORN2 has recently been implicated in that pathway, however, its action mechanism is unknown (27).

In several instances, LC3 conjugation onto the phagosome enhances the fusion between phagosome and lysosomes. Consequently, macrophages with defects in LAP pathway are less efficient in controlling intracellular growth of various bacteria such as *Legionella pneumophila, Staphylococcus aureus, M. tuberculosis, M. bovis* BCG, and *Listeria monocytogenes* (27, 28). Importantly, *in vivo* studies have shown that Rubicon-deficient mice have greater bacterial load and are more susceptible to *L. monocytogenes* infection (28). Another important function of LAP is to assist antigen presentation *via* MHC class II molecules, thus to bridge innate to adaptive immunity (24, 29). Of note, in some specific contexts, LC3 conjugation delays or does not affect at all phagosome maturation suggesting that LC3 alone is not sufficient to boost fusion with lysosomes (29, 30).

Autophagy machinery can also regulate phagocytosis indirectly by altering surface expression of phagocytic receptors. Lack of Atg protein Atg7 in macrophages results in upregulation of two class A scavenger receptors, MARCO and MSR1 that facilitate phagocytosis of *M. bovis* BCG and *M. tuberculosis* (31). Autophagy-deficient cells accumulate p62, which promotes dissociation of transcription factor nuclear erythroid-related factor 2 from Keap1 and then its translocation into the nucleus to mediate expression of these receptors. Notably, in another instance, autophagy deficiency can lead to reduced phagocytosis depending on the nature of the bacteria (32).

### Inflammation Dampening

Although inflammation is central to control bacterial infection, excessive inflammatory responses can lead to host tissue injury, and disease progression. Numerous studies have demonstrated the importance of autophagy in inflammation regulation in infectious and non-infectious settings, the interested readers can refer to recent reviews for a comprehensive view on this subject (3, 5). Here, we will only discuss on the beneficial role of autophagy machinery in modulating inflammation in a context of bacterial infections and macrophages. One key role of autophagy is the down-regulation of inflammasome activation through multiple mechanisms (33, 34). *Pseudomonas aeruginosa* infection results in mitochondrial damage that leads to NLRC4 inflammasome. Elimination of damaged mitochondria *via* autophagy, i.e., mitophagy, limits inflammasome activation both *in vitro* and *in vivo* (35). Similarly, in a *P. aeruginosa* septic model, *atg7^fl/fl^* mice have an enhanced susceptibility to infection with important neutrophil infiltration and severe lung damage. Loss of Atg7 in alveolar macrophages results in upregulation of IL-1beta and pyroptosis (36). Besides mitophagy, autophagy can also restrain inflammasome activation by capturing inflammasome subunits (37, 38). Lastly, autophagy can prevent non-canonical caspase-11 inflammasome activation by targeting *S. typhimurium* and thus limiting LPS release into the cytosol (39).

Finally, mechanisms and levels of autophagy dependent greatly on macrophage microenvironment. While IL-4, IL-13, and IL-10 appear to inhibit autophagy, proinflammatory cytokines such as IFNgamma, IL-1beta, and TNFalpha, activate this process (40). Specifically, IFNgamma-mediated xenophagy requires additionally ubiquilin-1 and guanylate-binding proteins 1 and 7 to promote recruitment of autophagy proteins p62 and Atg4B (41, 42). On another hand, the presence of vitamin D in serum enhances significantly macrophage autophagy *via* expression of cathelicidin antimicrobial peptide (43). Specific T cells can also stimulate autophagic activity in *M. tuberculosis*-infected human macrophages (44). Finally, microbiota may influence autophagy response too, as recently, probiotic *Bacillus amyloliquefaciens* has been shown to upregulate autophagy genes in macrophages, which lead to enhanced *Escherichia coli* killing (45).

## Bacterial Pathogens Evade Autophagy

**Figure 2:**
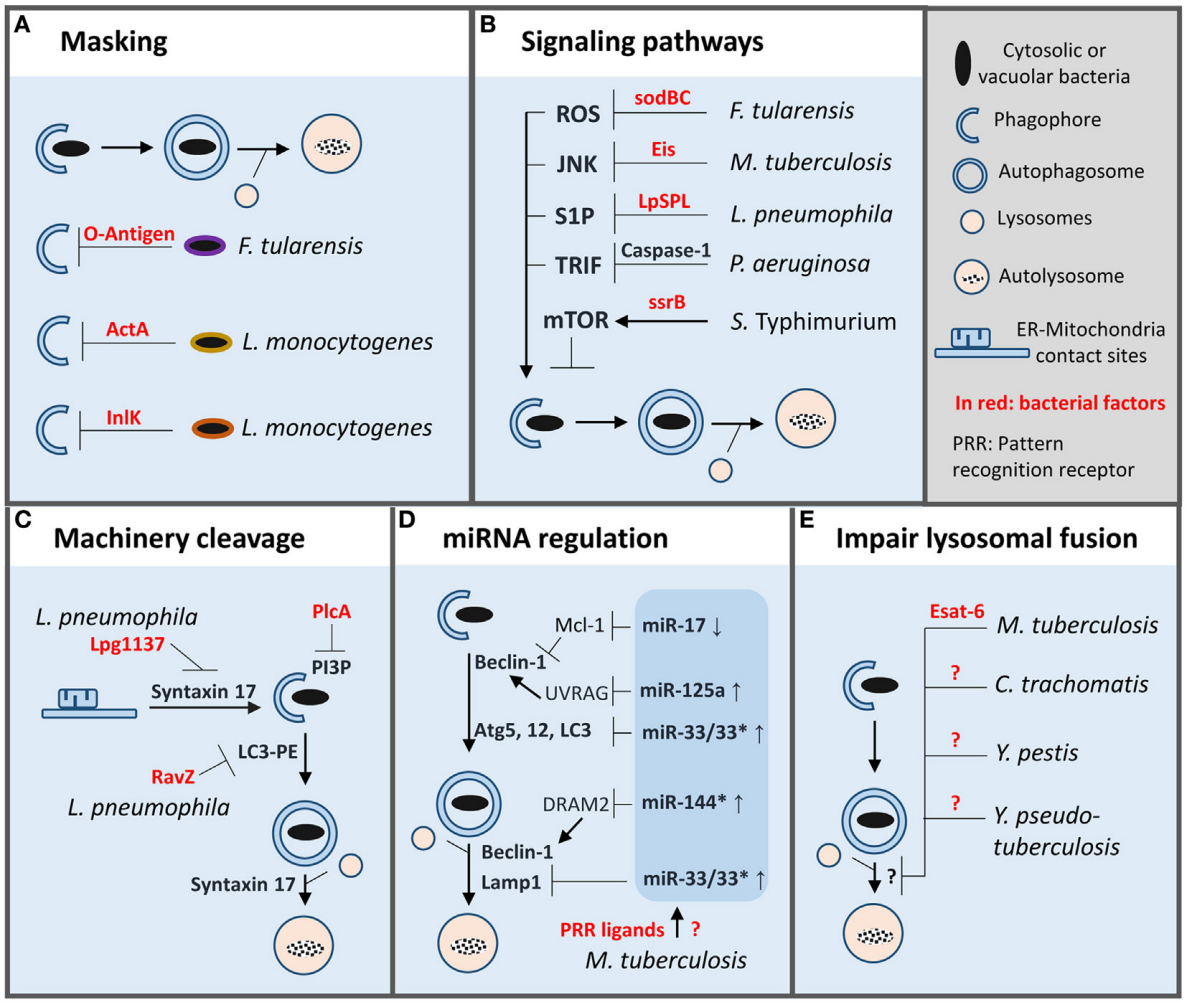
Autophagy evasion strategies adopted by bacterial pathogens inside macrophages. To evade autophagy in macrophages bacterial pathogens have developed a wide array of strategies such as: **(A)** masking of microbial surface to avoid recognition. **(B)** Manipulation of macrophage signaling pathways involved in autophagy regulation. **(C)** Direct cleavage of autophagy proteins or signaling lipid. **(D)** Limitation of autophagy machinery expression or function by miRNA regulation. **(E)** Inhibition of autophagosomes/lysosome fusion. PI3P, phosphatidylinositol 3-phosphate; LC3-PE, LC3 lipidated with phosphatidylethanolamine.

Intracellular bacterial pathogens have developed a wide array of tactics to counterbalance macrophage antibacterial defenses and autophagy is no exception (46). Most of the uncovered strategies are directed against xenophagy and target different steps of the process (Figure 2).

### Masking Bacterial Surface to Prevent Recognition

Cytosolic *L. monocytogenes* avoids recognition by autophagy machinery by secreting two virulence factors ActA and InlK (47, 48). These factors promote recruitment of host proteins, actin, and major vault protein, respectively, to form a protective coat that masks the bacteria. *Francisella tularensis* disguises itself directly by producing a surface polysaccharide, the O-antigen, which prevents cytosolic sensing of the pathogen and thus xenophagy (49). Importantly, cytosolic O-antigen mutants are killed by Atg5-dependent autophagy inside murine macrophages.

### Regulation of Signaling Pathways Involved in Autophagy Initiation

In macrophages, *S. typhimurium* prevents autophagy by activating mTOR, a master repressor of autophagy (50, 51). Two mechanisms seem to be at play, first, the degradation of the energy sensor, Sirt1/AMPK complex, which negatively regulates mTOR resulting in autophagy activation and, second, the recruitment of non-receptor tyrosine kinase focal adhesion kinase (FAK), an activator of Akt/mTOR pathway. SsrB, a response regulator of a two-component system involved in the regulation of SPI2 encoded virulence factors, promotes AMPK down-regulation (50). Notably, *in vivo*, macrophages deficient in FAK are more efficient in clearing *S. typhimurium* infection than their wild-type counterpart (51).

Conversely, some pathogens can inhibit signaling pathways that promote autophagy. *M. tuberculosis* secretes an *N*-acetyltransferase, Eis, to target JNK-dependent autophagy; however, it does not seem to be sufficient to affect intracellular growth (52, 53). In addition, *M. tuberculosis* limits LAP by inhibiting NADPH oxidase recruitment onto its phagosome, which favors pathogen intracellular growth *in vitro* and *in vivo* (54). Interestingly, antioxidant enzymes SodB and SodC of *F. tularensis* prevent ROS-induced xenophagy and possibly LAP (55). Further, vacuolar *L. pneumophila* translocates a sphingosine-1 phosphate lyase into the cytosol to alter sphingosine metabolism implicated in autophagy activation (56). Lastly, some pathogens can take advantage of negative feedback regulatory circuits present in macrophages to target autophagy. *P. aeruginosa* infection leads to NLRC4-dependent caspase-1 activation which results in cleavage of TRIF, an important mediator of TLR4-induced autophagy (57). Importantly, *in vivo*, preventing TRIF cleavage restores autophagy and bacterial clearance.

### Cleavage of Autophagy Machinery

*L. pneumophila* can also block autophagy directly by secreting two specific proteases. First, Lpg1137 targets ER-mitochondria contact sites to cleave syntaxin-17, a key SNARE protein implicated in autophagosome formation (58, 59). On another hand, RavZ, a cysteine protease, cleaves phosphatidylethanolamine-conjugated LC3 to produce a permanent unlipidated form of LC3 that is inadequate for the formation of autophagosomes (60). Another example is *L. monocytogenes*, which secretes phospholipase PlcA to degrade PI3P, a key lipid produced by Beclin-1 complex, and involved in LC3 lipidation (48, 61).

### Regulation of miRNA (miR) Targeting Autophagy Machinery

Besides post-translational modification, autophagy can also be modulated at a post-transcriptional level *via* expression of various small non-coding RNAs (miR). *M. tuberculosis* thwarts autophagy by inducing expression of miR-33 which down-regulates expression of several autophagy proteins along the pathway (Atg5, Atg12, LC3, and LAMP1) (62). Remarkably, mice with hematopoietic miR-33 deficiency have a superior capacity to control *M. tuberculosis* infection. Another miR induced by *M. tuberculosis*, is miR-125a, which targets UVRAG in complex with Beclin-1 to inhibit autophagy and thus promote pathogen intracellular survival inside macrophages (63). Interestingly, miR-33 and miR-125a can be induced by PRR ligands isolated from *M. tuberculosis* (62, 63). Expression of these miR may be a normal negative feedback loop present in host cells to prevent excessive autophagy, which *M. tuberculosis* exploits to its advantage. Additionally, *M. tuberculosis* upregulates miR-144* to reduce expression of DRAM2, a recently discovered autophagy protein, that promotes activation of a second Beclin-1 complex involved in autophagosome maturation (64). Finally, *M. tuberculosis* can down-regulate miR-17, which targets Mcl-1, an inhibitor of Beclin-1 complex involved in autophagy initiation (65).

### Blockade of Autophagosome–Lysosome Fusion

As mentioned above, *M. tuberculosis* can block autophagosome maturation *via* miR-144*, although, the bacterial factor(s) involved in that process remain to be identified (65). Separate studies have shown that Esat-6, an important *M. tuberculosis* virulence factor, plays also a role in blocking autophagosome maturation in macrophages as well as in dendritic cells (66, 67). The action mechanism of Esat-6 is unknown but might be mediated, in part, by miR modulation (68). Several other bacterial pathogens can impair autophagosome maturation for survival or growth in macrophages such as *Chlamydia trachomatis, Yersinia pestis, Y. pseudotuberculosis*, but like for *M. tuberculosis* the underlying molecular mechanisms are unknown (69–71). Furthermore, it is unclear whether these mechanisms applied to both xenophagy and LAP.

## Autophagy Machinery Favors Bacterial Pathogens

### Nutrient and Membrane Acquisition

While some pathogens neutralize autophagy to replicate intracellularly, others exploit autophagy or some autophagy proteins to acquire nutrients and remodel their vacuoles. *Y. pseudotuberculosis* inhibits autophagosome maturation; however, it relies on autophagosome formation for its growth in macrophages (71). An early study has shown that exogenous autophagy activation promotes the development of *Coxiella*-replicative vacuole (72). In macrophages and epithelial cells, *C. burnetti* resides in an large acidified compartment, which fuses with mature autophagosomes *via* Cig2, a type IV secretion system effector (73, 74). Interestingly, *in vivo*, Cig2 reduces host tolerance to *C. burnetti* infection without affecting bacterial load (74).

*Brucella abortus* subverts ULK1 and Beclin-1 complexes to remodel its ER-derived vacuole into a compartment with autophagic properties (75). Nonetheless, this conversion is independent of the two ubiquitin-like conjugation systems, Atg5-12 and LC3. This selective manipulation of autophagy proteins is important for *Brucella* lifecycle and cell-to-cell spreading. Similarly, *M. marinum*, a species closely related to *M. tuberculosis*, uses autophagy machinery to promote bacterial egress from its natural host, *Dictyostelium*, a macrophage-like unicellular organism (76). How *Brucella* and *M. marinum* manipulate autophagy proteins to favor transmission is still unknown.

Some pathogens have a more complex relationship with autophagy in a sense that they can evade, as outlined above, or exploit autophagy depending on the infection stages or the type of autophagy. At early stage, vacuolar *Legionella* interacts with autophagy pathway and seems to rely on autophagosome for survival in permissive macrophages (77). Similarly, cytosolic *F. tularensis* triggers an Atg5-independent autophagy pathway to acquire nutrients and replicate while avoiding xenophagy (78). The molecular differences between these two autophagy-related pathways, one that favors bacterial growth and the other promotes killing, definitely call for further investigation. Finally, immediately after entering macrophages, *L. monocytogenes* manipulates LAP to form spacious non-acidified *Listeria*-containing phagosomes (SLAPs) which are believed to participate in persistent infection (79, 80). Low expression of virulent factor, Listeriolysin O, appears to play a role in SLAP formation whereas high-expression triggers bacterial escape into the cytosol (80).

### Inflammation Dampening

One example is *Vibrio parahaemolyticus*, which limits NLRC4 inflammasome-mediated IL-1beta production by activating macrophage autophagy *via* a type III secretion effector, VopQ (81). Other bacteria seem to benefit from the autophagy machinery in a more passive way. For example, Atg16L1-deficient mice clear more efficiently uropathogenic *E. coli* (UPEC) and *Citrobacter rodentium* than wild-type controls (82–84). The improved protection against *C. rodentium* is associated with an enhanced immune response dependent on monocytes; however, it does not rely on a cell-intrinsic role of Atg16L1 in myeloid cells (84). In contrast, loss of Atg16L1 in macrophages is responsible for the phenotype observed during UPEC infection (83). Atg16L1-deficient macrophages, infected with UPEC, activate more NLRP3/Caspase-1 inflammasome, and IL-1beta production. Importantly, *in vivo*, IL-1beta neutralization reduces the capacity of Atg16L1-deficient mice to control UPEC infection. Testing other autophagy proteins will be essential to confirm the role of autophagy entire pathway in that process.

## Autophagy in Host-Directed Therapy (HDT) and Vaccine

### Host-Directed Therapy

In the past few years, a number of studies have highlighted the potential of targeting autophagy for the control of bacterial infections. FDA-approved drugs with proautophagy activity, such as statin, gefitinib, carbamazepine, and metformin, have been shown to limit *M. tuberculosis* growth in mice model of infection (85–88). Similarly, rapamycin enhances clearance of *P. aeruginosa* and *B. cepacia, in vivo*, in addition to reducing lung inflammation (89, 90). However, these drugs can module several other cellular functions, so it is unclear whether their action, *in vivo*, is mediated by autophagy. Another difficulty is the seemingly opposing effects of a same drug depending on the infection settings. Indeed, while rapamycin induces *M. tuberculosis* killing in macrophages, it promotes bacterial survival in endothelial cells and in HIV-coinfected macrophages (91–93). The only compound tested, *in vivo* that specifically activates autophagy is a small cell permeant peptide, TAT-Beclin-1 (94). This designed peptide has been shown to improve outcome of chikungunya and West Nile virus infections in mice, thus it would be a good candidate to test in the context of bacterial infection. However, one has to bear in mind that some pathogens use autophagy to thrive in host cells and other can either evade or exploit this pathway according to the infection stage. Therefore, a detailed knowledge of the role(s) of autophagy and its molecular mechanisms for each bacterial pathogen is mandatory to develop more specific autophagy modulators, as well as, the availability of relevant preclinical models to evaluate the efficacy of these compounds.

### Vaccine

Manipulation of autophagy in antigen-presenting cells including macrophages holds also great promise in vaccine development. Autophagy plays an important role in antigen processing and MHC presentation (95). Studies already indicate that boosting autophagy can enhance antigen presentation and vaccine efficacy. For instance, mice immunized with BCG-infected dendritic cells treated with mTOR inhibitor, rapamycin, have greater Th1-mediated protection after being challenged with *M. tuberculosis* (96). Recently, BCG*ΔureC::hly*, a live vaccine with improved immunogenicity and in clinical trial phase II, has been shown to enhance xenophagy (97). However, the importance, *in vivo*, of phagocyte autophagy in vaccine enhanced-efficacy is unclear as both cited strategies trigger other cellular pathways.

## Conclusion

Macrophage autophagy is central to host defenses against bacterial infections, sending intracellular pathogens to lysosomes for degradation while controlling inflammation to limit host damages. Since novel functions for autophagy proteins have emerged in various physiological and pathological situations, it is likely that further contributions of autophagy machinery to macrophage biology will be unveiled in the context of bacterial infection (98, 99). On the other hand, pathogens have found multiple subterfuges to manipulate this machinery in order to persist or proliferate, still, their action mechanisms and significance *in vivo* are far from being thoroughly understood. A more comprehensive and integrated view of bacteria-autophagy interplay will definitely help in designing more specific HDT and vaccine based on autophagy modulation.

## Author Contributions

IV and AB reviewed the relevant literature and wrote the manuscript. IV prepared and AB revised the figures. IV and AB have read and approved the final manuscript.

## Conflict of Interest Statement

The authors declare that the research was conducted in the absence of any commercial or financial relationships that could be construed as a potential conflict of interest.
